# Evaluation of computed tomography images under deep learning in the diagnosis of severe pulmonary infection

**DOI:** 10.3389/fncom.2023.1115167

**Published:** 2023-08-04

**Authors:** Mao Ming, Na Lu, Wei Qian

**Affiliations:** ^1^Department of Infectious Disease, South of Guang’anmen Hospital, China Academy of Chinese Medical Sciences, Beijing, China; ^2^Department of Colorectal Surgery, South of Guang’anmen Hospital, China Academy of Chinese Medical Sciences, Beijing, China; ^3^Department of Intensive Care Unit, South of Guang’anmen Hospital, China Academy of Chinese Medical Sciences, Beijing, China

**Keywords:** empty convolution, deep convolutional neural network, severe pneumonia, pulmonary infection, computed tomography images

## Abstract

This work aimed to explore the diagnostic value of a deep convolutional neural network (CNN) combined with computed tomography (CT) images in patients with severe pneumonia complicated with pulmonary infection. A total of 120 patients with severe pneumonia complicated by pulmonary infection admitted to the hospital were selected as research subjects and underwent CT imaging scans. The empty convolution (EC) and U-net phase were combined to construct an EC-U-net, which was applied to process the CT images. The results showed that the learning rate of the EC-U-net model decreased substantially with increasing training times until it stabilized and reached zero after 40 training times. The segmentation result of the EC-U-net model for the CT image was very similar to that of the mask image, except for some deviations in edge segmentation. The EC-U-net model exhibited a significantly smaller cross-entropy loss function (CELF) and a higher Dice coefficient than the CNN algorithm. The diagnostic accuracy of CT images based on the EC-U-net model for severe pneumonia complicated with pulmonary infection was substantially higher than that of CT images alone, while the false negative rate (FNR) and false positive rate (FPR) were substantially lower (*P* < 0.05). Moreover, the true positive rates (TPRs) of CT images based on the EC-U-net model for patchy high-density shadows, diffuse ground glass density shadows, pleural effusion, and lung consolidation were obviously higher than those of the original CT images (*P* < 0.05). In short, the EC-U-net model was superior to the traditional algorithm regarding the overall performance of CT image segmentation, which can be clinically applied. CT images based on the EC-U-net model can clearly display pulmonary infection lesions, improve the clinical diagnosis of severe pneumonia complicated with pulmonary infection, and help to screen early pulmonary infection and carry out symptomatic treatment.

## 1. Introduction

Inflammation in lung tissues (bronchioles, alveoli, and interstitium) caused by various etiologies and pathogens on different occasions has similar or the same pathophysiological process and can deteriorate into severe pneumonia (Zhou et al., 2021; Al Khoury et al., 2022). It can be caused by various pathogenic causes. Pneumonia with cardiopulmonary foundation or additional risk factors or infection with special pathogenic microorganisms, such as severe acute respiratory syndrome (SARS) virus, avian influenza virus, and legionella bacteria, will aggregate pneumonia and increase the risk of death (Cai and Zheng, 2020; Gerges Harb et al., 2020; Wan et al., 2021). Severe pneumonia is a serious respiratory disease, and most patients will be complicated with organ dysfunction. In addition to the common respiratory symptoms of pneumonia, there are respiratory failure and obvious involvement of the circulatory system, nervous system, and other systems. Common symptoms include fever, chills, cough, expectoration, chest pain, dyspnea, and increased respiratory rate (Issa et al., 2020). Severe pneumonia will result in various sequelae, the most common of which is lung injury (such as bullae, empyema, and pyopneumothorax) and heart-related diseases, including heart failure or pulmonary heart disease. Therefore, it is very important to pay attention to the early diagnosis and treatment of severe pneumonia.

Imaging examination is an important process in the diagnosis of pneumonia and is one of the important indexes to judge severe pneumonia. Clinical diagnosis of lung lesions often adopts X-ray, bedside ultrasound, conventional chest computed tomography (CT) plain scan, etc (Hu et al., 2021). Chest X-ray examination is relatively convenient and cost-effective, but it exhibits great limitations in the patient’s position and scope of fluoroscopy, which limits the imaging results and easily leads to a false negative result (Sayad et al., 2021). Ultrasound shows the lungs clearly and features with low price, is easy to operate, and is easily disturbed by lung gas. CT images are grayscale images with high density resolution that can clearly display the lung and other soft tissue organs at low cost and have been widely used in the diagnosis of lung diseases.

Image segmentation refers to finding and distinguishing the target area according to the properties and characteristics of the image. In the medical field, segmenting the images of tissue and organ lesions is an important auxiliary means for clinical diagnosis, treatment, and efficacy evaluation of diseases. Traditional image segmentation algorithms are still widely used, even in commercial applications. However, with the exponential growth of the current data volume, the requirements for the depth of information mining and segmentation technology are increasing, so it is necessary to study higher-level technologies (Arej et al., 2022). Deep learning is a deep nonlinear structure that is based on the human neural network mechanism, layered feature extraction, and recognition. Ideally, as long as the amount of data is sufficient and the network is deep enough, an ideal effect can be achieved, and the accuracy rate of human beings can even be exceeded (Diab et al., 2020; Alimoradi et al., 2021). Due to its excellent quality, deep learning is also widely used in medical image processing. Therefore, deep learning was combined with CT imaging technology and applied in clinical diagnosis in this work. Wang et al. (2021) discussed the application of deep learning technology in conical beam computed tomography image analysis of oral lesions, and processed images by artificial segmentation, threshold segmentation algorithm, and full convolutional neural network algorithm. The results showed that the image segmentation accuracy of the full convolutional neural network algorithm was superior to the traditional manual segmentation and threshold segmentation algorithms. Wu et al. (2020) proposed a deep convolutional neural network fusion support vector machine algorithm (DCNN-F-SVM) and applied it to brain tumor image segmentation. According to the segmentation results obtained, the image segmentation performance of this model was significantly better than that of deep convolutional neural network and integrated SVM classifier.

In summary, the combination of deep learning technology and medical imaging is still the focus of clinical research. Therefore, 120 patients with severe pneumonia complicated with pulmonary infection were selected as subjects for CT imaging scanning. An EC-U-net network model based on empty convolution (EC) and the U-net network phase was constructed and applied to patient CT image processing. The diagnostic value of a deep convolutional neural network (CNN) combined with CT images for severe pneumonia complicated with pulmonary infection was discussed by analyzing the imaging characteristics of patients. In this study, deep learning technology was innovatively combined with lung CT image, which was jointly applied in clinical treatment, providing a theoretical reference for the evaluation of lung infection in patients with pneumonia.

## 2. Materials and methods

### 2.1. Research objects

In this work, 120 patients with severe pneumonia complicated with pulmonary infection, aged 20–69, admitted to the hospital from November 2019 to April 2021, were selected as the research subjects. This study was approved by the medical ethics committee of the hospital, and patients and their families were informed of this study and signed informed consent.

Inclusion criteria: (i) patients older than 18 years; (ii) patients with complete clinical data; (iii) patients who signed informed consent; (iv) patients who met the diagnostic criteria for severe pneumonia formulated by the American Society of Infectious Diseases/American Thoracic Society in 2007 (Vetrugno et al., 2020); and (v) the diagnosis of severe pneumonia was in accordance with the guidelines of the Respiratory Society of Chinese Medical Association in 2006 (Kim, 2020).

Exclusion criteria: (i) patients with autoimmune diseases; (ii) patients complicated with organ transplantation; (iii) patients with other tumors; (iv) patients with heart disease and other important organ damage; and (v) patients who had poor compliance with examination.

### 2.2. CT image scanning

All patients were scanned by 64-row spiral CT. The patients were placed in the supine position and scanned from the chest entrance to the bottom of the lung. The scanning parameters were as follows: layer thickness of 2.5 mm, pitch of 1.25, tube voltage of 120 kV, tube current of 120 mA, and matrix of 521 × 521.

### 2.3. CT image segmentation based on the deep learning model

**FIGURE 1:**
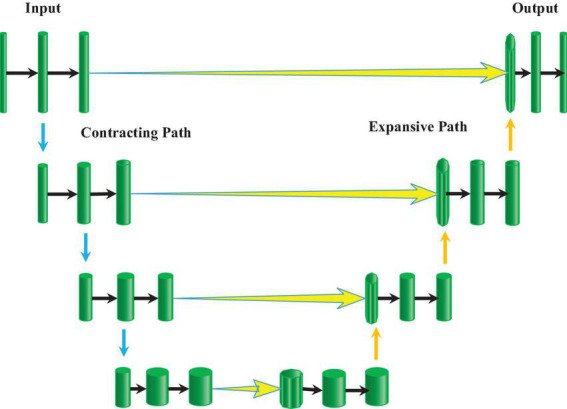
Schematic diagram of the U-net network structure.

The U-net model (Feng et al., 2020) is an improved fully convolutional network (FCN) structure, which is generally composed of a contracting path on the left half and an expansive path on the right half (Figure 1). The compression channel is a typical CNN structure. It repeats the structure with two convolutional layers and one maximum pooling layer. The dimensionality of the feature map is doubled after each pooling operation. In the expansion channel, a deconvolution operation was performed first to reduce the dimensionality of the feature map by half, and then the feature maps obtained from the corresponding compression channel were spliced to reconstitute a feature map of two times the size. Then, two convolutional layers were adopted for feature extraction, and this structure was repeated. In the final output layer, two convolutional layers were employed to map the 64-dimensional feature map into a 2-dimensional output map.

**FIGURE 2:**
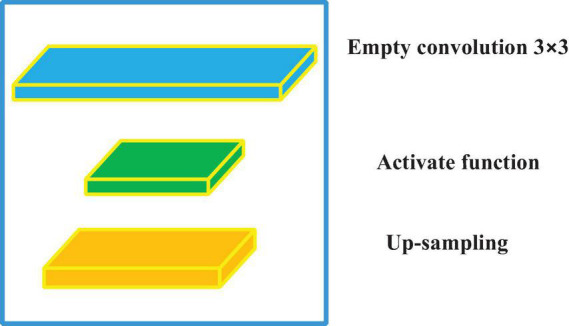
The convolution blocks of the EC-U-net network model.

Empty convolution (EC) (Moore and Gardiner, 2020) is essentially a convolution with intervals. It can enlarge the receiving field without changing the number of parameters and enhance the ability of the model to extract information. EC and the U-net network were combined to design an EC-U-net network model in this work. The convolution block of the model (Figure 2) mainly included the EC and activation function.

In the field of mathematics, convolution is an operation on a function. In fact, it is a weighted summation process, which is an integral operation. The convolution operation is expressed as the following equation.


(1) $$\left(h_{1}^{*}\,h_{2}\right)\,\left(t\right)≜\int_{-\infty }^{\infty }h_{1}\,\left(\upsilon \right)\,h_{2}\,\left(t-\upsilon \right)\,d\upsilon$$

In equation (1), *h*_*1*_ and *h*_*2*_ are functions, and two continuous functions are integrable within the real number range. When CNN is adopted to process the CT image, image pixels are used as input, the convolution kernel is an impact function that acts on the system and can extract system features, and the output is a feature map corresponding to the image. Therefore, the image convolution process is actually a linear operation, and the convolution of a two-dimensional vector is expressed as follows.


(2) $$P^{*}\,\left(i,j\right)=\left(P\times C\right)\,\left(i,j\right)=\sum_{a}\sum_{b}P\,{\left(a+i,b+j\right)}^{*}\,C\,\left(a,b\right)$$

In equation (2), *P* represents the input, *C* represents the convolution kernel, with a step size of 1, P* represents the output, and (*i*, *j*) represents the pixel coordinates. Since the input data dimension may not be an integer multiple of the convolution kernel dimension, the method of padding zeros in the edge area is usually adopted to protect effective information, and the filling column is introduced.


(3) $$P=\left[\frac{i-c+2\,k}{l}\right]+1$$

In equation (3), *k* represents the filling column, and *l* represents the step size. Weight sharing is a major feature of CNNs, which can greatly reduce the number of parameters and increase the nonlinearity of the model. Then, the total number of parameters (TNP) is calculated as shown in the following equation.


(4) $$T\,N\,P=m+n=c^{2}*z*j+j$$

In equation (4), *m* represents the weight, *n* represents the bias value, *z* is the number of feature channels, and *j* represents the feature map. In practical applications, the EC may cause some image pixels to not participate in the convolution calculation due to the interval, thereby losing the continuity of some information. To solve this problem, the hole size of the model network is designed according to the hybrid dilated convolution (HDC) standard. The following condition is needed.


(5) $$T_{i}=\max\left[T_{i+1}-2\,e_{i},T_{i+1}-2\,\left(T_{i+1}-e_{i}\right),e_{i}\right]$$

In equation (5), *e*_*i*_ is the space interval of the *i*-th layer, and *T*_*i*_ is the space interval of the *i*-th layer.

Calculation of the convolutional layer is essentially a linear weighted summation, so the model lacks nonlinear expression, and the expression ability is extremely limited. Therefore, an activation function should be introduced. In this work, the sigmoid function (Ma et al., 2021) is used for classification output, and the ReLU function (Kwee and Kwee, 2020) is employed for internal feature extraction.

**FIGURE 3:**
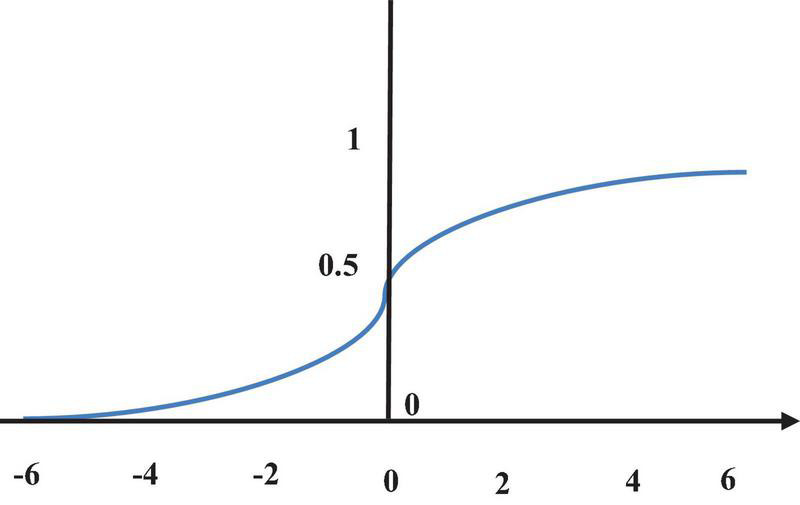
Sigmoid function.

The sigmoid function can compress the value of the function to the range of (0, 1), and it can be derived everywhere (Figure 3), which is expressed as follows.


(6) $$\text{Sigmoid}=1\,/\,\left(1+e^{-\left(m\,x+n\right)}\right)$$

The sigmoid function is related to the parameter update and model optimization, and the step size of the parameter update is related to the gradient. The reverse transfer process of the gradient between the layers can be expressed as the following equation.


(7) $$\frac{\partial D}{\partial b_{u}}={\text{Sigmoid}}^{\prime }\,\left(z_{c}\right)\,m_{c+1}\,{\text{Sigmoid}}^{\prime }\,\left(z_{c+1}\right)\,m_{c+2}$$

$$\cdots \,{\text{Sigmoid}}^{\prime }\,\left(z_{u}\right)\,\frac{\partial D}{\partial a_{v}}$$

In equation (7), *a*_*v*_ is the *v*-layer output, and $\frac{\partial D}{\partial b_{u}}$ is the gradient of the objective function to the bias term.

**FIGURE 4:**
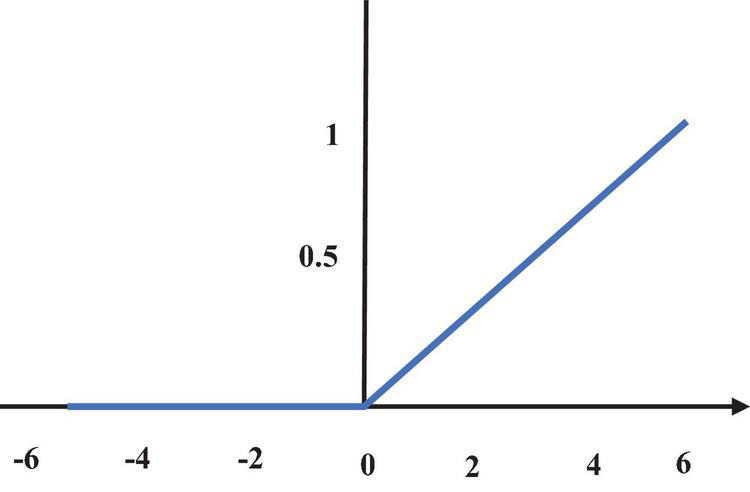
ReLU function diagram.

The ReLU function (Figure 4) can effectively avoid gradient disappearance. It is an optimization of the sigmoid function, which is expressed as the following equation.


(8) R⁢e⁢l⁢u⁢()=max⁡(0,z)

(9) $$R\,e\,l\,u\,{\left(\right)}^{\prime }=\left\{ \begin{array}{cc} 0 & z<0\\ 1 & z>0 \end{array}\right.$$

After the model is constructed, a learning criterion should be set to supervise the model or select the optimal model. The cross-entropy loss function (CELF) and Dice coefficient are used as the learning criteria, which are expressed as the following equations.


(10) $$C\,E\,L\,F\,\left(\right)=-\frac{\sum \left[X\,\log\left(F\,\left(I\right)\right)+\left(1-X\right)\,\log\left(F\,\left(I\right)\right)\right]}{n}$$

(11) $$D\,i\,c\,e=2^{*}\,\frac{M\cap N}{\left|M\right|+\left|N\right|}$$

In equation (11), *M* represents the pixel matrix of the image mask, *N* is the pixel matrix of the output predicted image, and *M* ∩ *N* represents the inner product of the two image matrices.

### 2.4. Construction of the experimental environment

The operating system is Windows 10, the processor uses Xeon CPU E5-2630, and the graphics card uses NVIDIA Quadro K2200. The framework uses TensorFlow, the language uses Python3.5, and the dependent libraries use CUDA9.0, cudnn, OpenCV, and SimpleITK.

The data set uses lung data from the Kaggle competition, which includes 2,650 lung images and corresponding 250 mask images made by experts. The training set and test set are set to 1:1.

### 2.5. Statistical methods

SPSS 19.0 was used for data processing in this study. The mean ± standard deviation ($\bar{\text{X}}$ ± s) was used to indicate the measurement data, and the percentage (%) was used for counting data. Pairwise comparisons were performed by one-way ANOVA. The difference was statistically significant at *P* < 0.05.

## 3. Results

### 3.1. Experimental results

**FIGURE 5:**
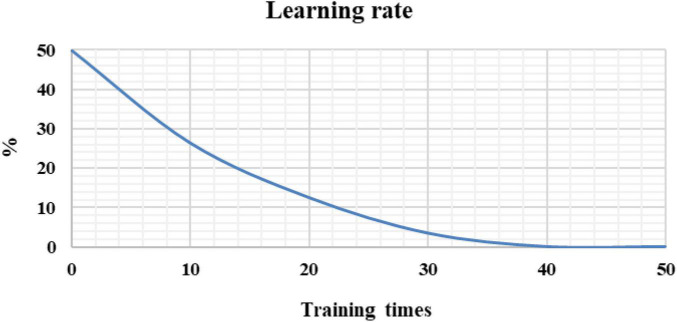
Model learning rate under different training times.

In Figure 5, the learning rate of the EC-U-net model decreased substantially as the number of training iterations increased until it stabilized and became zero after 40 training iterations.

**FIGURE 6:**
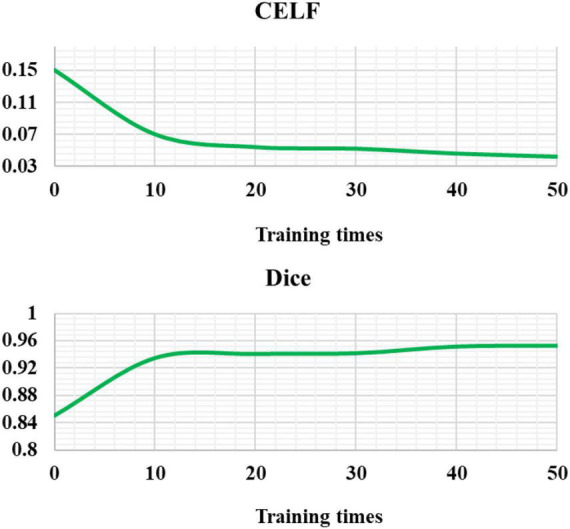
Cross-entropy loss function and Dice coefficient of the EC-U-net model.

The CELF and Dice coefficients were compared (Figure 6). The CELF of the EC-U-net model attenuated with increasing training times, while the Dice coefficient increased with increasing training times (gradually approaching 1) until it was stable.

### 3.2. Application effect of the EC-U-net model in CT images

**FIGURE 7:**
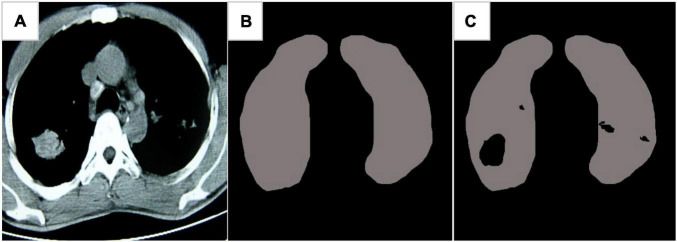
Image segmentation results of the EC-U-net model. **(A)** Lung CT; **(B)** mask diagram; **(C)** segmentation results.

Figure 7 showed the CT image segmentation result of the EC-U-net model. The result of CT image segmentation using the EC-U-net model was very similar to the mask image, but there were some deviations in the segmentation at the edge.

**FIGURE 8:**
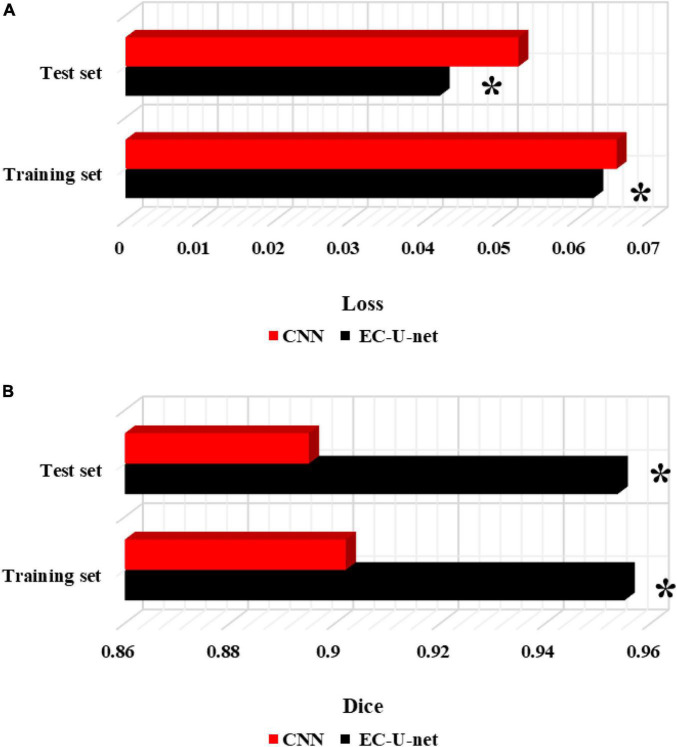
Comparison of segmentation performance between the traditional CNN algorithm and this model. **(A)** CELF; **(B)** Dice coefficient. *Compared to the CNN algorithm, *P* < 0.05.

The traditional CNN algorithm was introduced and compared with the segmentation results of the established model (Figure 8). The CELF of the EC-U-net model for lung CT image segmentation was observed to be substantially smaller than that of the CNN algorithm, and the difference was considerable (*P* < 0.05). The Dice coefficient of the EC-U-net model for lung CT image segmentation was substantially greater than that of the CNN algorithm (*P* < 0.05).

### 3.3. Patient imaging findings

**FIGURE 9:**
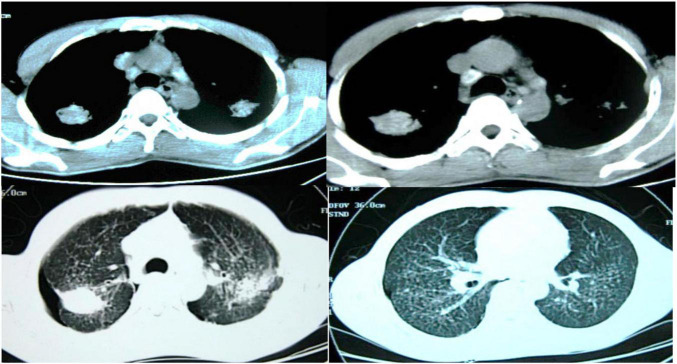
CT images of a 38-year-old male patient.

Figure 9 showed the CT images of a 38-year-old male patient, showing multiple small nodules in both lungs, mostly in the upper and posterior parts of the lungs; fibrotic masses were observed in the posterior segments of the upper lobes of both lungs, with bilateral symmetry and extravasation-like changes. Pulmonary bullae were observed below the pleura in the periphery of the lungs where the nodules were concentrated, and a pneumothorax shadow was seen on the periphery of the lungs with localized pleural hypertrophy.

**FIGURE 10:**
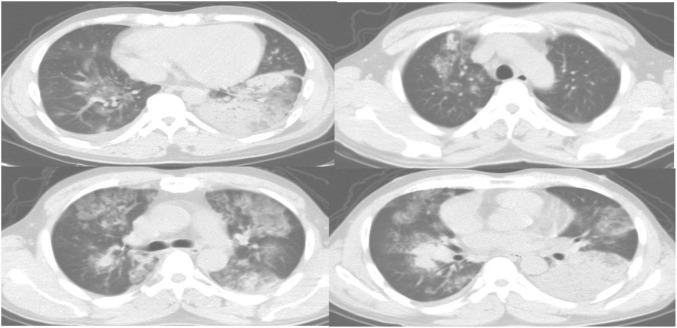
CT images of a 51-year-old male patient.

Figure 10 showed the CT images of a 51-year-old male patient, showing multiple segmental lesions in both lungs spreading more than before. It was considered infectious lesions, multiple small lymph nodes in the mediastinum, and a small amount of free effusion in the right pleural cavity.

### 3.4. Comparison of patient diagnosis accuracy, false positive rate (FPR) and false negative rate (FNR)

**FIGURE 11:**
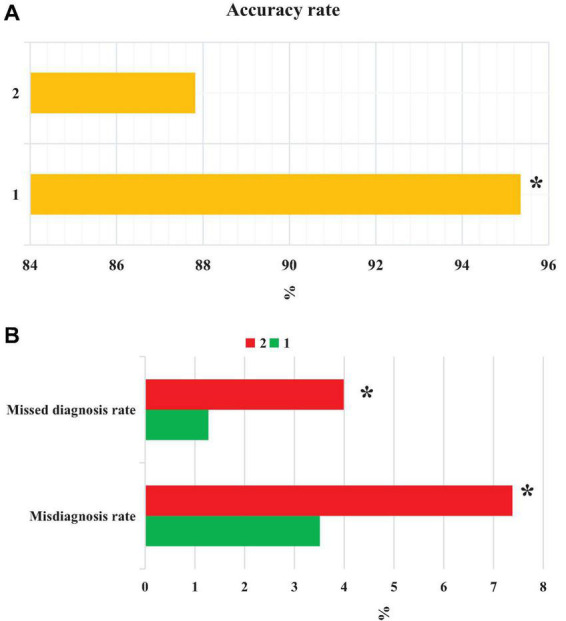
Comparison of patient diagnosis accuracy, FNR, and FPR. **(A)** accuracy; **(B)** FNR and FPR. 1: CT image based on the EC-U-net model; 2: original CT image. *Compared with 1, *P* < 0.05.

From Figure 11, the accuracy of CT images based on the EC-U-net model in the diagnosis of severe pneumonia combined with infection was substantially higher than that of CT images (*P* < 0.05). The FPR and FNR of CT images based on the EC-U-net model for severe pneumonia complicated by infection were substantially lower than those of CT images, and the differences were considerable (*P* < 0.05).

### 3.5. Comparison of diagnosis results of CT imaging features

**FIGURE 12:**
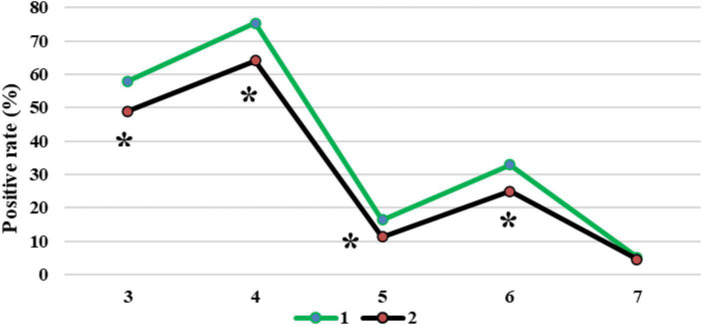
Comparison of diagnosis results of patients with CT imaging features. 1: CT image based on the EC-U-net model; 2: original CT image; 3: patchy high-density shadow; 4: diffuse ground-glass-like density shadow; 5: pleural effusion; 6: lung consolidation shadow; 7: reticular nodule shadow. *Compared with 1, *P* < 0.05.

Figure 12 compared the diagnosis results of patients with CT imaging features. The CT image based on the EC-U-net model had a true positive rate (TPR) of 57.93% for patchy high-density shadows and a TPR of 75.31% for diffuse ground-glass density shadows. The TPRs were 16.39, 32.88, and 5.08% for pleural effusion, pulmonary consolidation, and reticular nodules, respectively. The original CT image had a TPR of 48.89% in the diagnosis of patchy high-density shadows, 64.03% in diffuse ground-glass density shadows, 11.27% in pleural effusion, 24.91% in pulmonary consolidation, and 4.55% in reticular nodules. In short, CT images processed by the EC-U-net model had a higher TPR for patchy high-density shadows, diffuse ground glass density shadows, pleural effusions, and lung consolidation shadows than the original CT images, and the differences were substantial (*P* < 0.05).

## 4. Discussion

Severe pneumonia is a very common critical symptom around the world. It usually occurs in elderly individuals. Because its onset is relatively insidious and there are no obvious symptoms in the early stage, it will lead to delayed detection of the patient’s condition and endanger the life of the patient (Salerno et al., 2021). Therefore, early examination and early treatment are of great significance to patients with severe pneumonia complicated with pulmonary infection (Ding et al., 2020; Huang et al., 2021). Thanks to the continuous development of computer technology, medical imaging technology has gradually exceeded the scope of traditional X-ray photography, among which CT imaging technology is widely adopted in the diagnosis of various diseases because of its high accuracy, low cost, and convenient operation. A total of 120 patients with severe pneumonia combined with pulmonary infection were selected as the research subjects and underwent CT imaging scans. Then, an EC-U-net network model was constructed based on empty convolution and the U-net network and applied to CT image processing. First, analysis of the performance of the model suggested that the learning rate of the EC-U-net model decreased substantially as the training times increased until it stabilized, and it even became 0 when there were 40 training times. Such results indicated that the training efficiency of the model was high and local fluctuations were avoided. The segmentation result of the CT image by the EC-U-net model was very similar to the mask image, but there were some deviations in the edge segmentation. This was different from the results of Shi et al. (2021), indicating that the segmentation effect on the microstructure of the lungs in the EC-U-net model was not satisfactory. The segmentation results of the introduced traditional CNN algorithm and the proposed model were compared. It was found that the EC-U-net model for lung CT image segmentation exhibited a substantially smaller CELF and a greatly larger Dice coefficient than the CNN algorithm (*P* < 0.05). This showed that the overall segmentation performance of the EC-U-net model for CT images was better than that of traditional algorithms, and it had clinical application feasibility (Gordaliza et al., 2018).

Accuracy reflects the precision of prediction. The FNR and FPR are a pair of indicators from the perspective of prediction coverage. The EC-U-net model was applied to the CT image processing of 120 cases of severe pneumonia combined with pulmonary infection. It was found that the accuracy of CT images based on the EC-U-net model in the diagnosis of severe pneumonia complicated by infection was substantially higher than that of CT images. The FNR and FPR of CT images based on the EC-U-net model for severe pneumonia complicated by infection were substantially lower than those of CT images, and the differences were great (*P* < 0.05). This showed that the combination of the EC-U-net model and CT images can effectively improve the clinical diagnosis of severe pneumonia complicated by pulmonary infection, improve the diagnostic accuracy, and help screen early pulmonary infections for symptomatic treatment (Haas et al., 2017; Borodulina et al., 2020). Then, the CT image characteristics of patients were analyzed, and the CT images based on the EC-U-net model had a higher TPR for patchy high-density shadows, diffuse ground-glass density shadows, pleural effusions, and pulmonary consolidation shadows, and the differences were notable (*P* < 0.05). This is similar to the research results of Morris et al. (2020), indicating that CT images based on the EC-U-net model can clearly show pulmonary infection lesions and determine the scope of the lesion, thereby providing a diagnostic basis for the early diagnosis of severe pneumonia combined with pulmonary infection.

## 5. Conclusion

In this research, 120 patients with severe pneumonia complicated with pulmonary infection were recruited to receive CT imaging scans. Furthermore, an EC-U-net network model based on the EC and U-net network phases was constructed and applied to process the CT images of patients. The results showed that the EC-U-net model was superior to the traditional algorithm in the overall performance of CT image segmentation and had feasibility for clinical application. CT images based on the EC-U-net model can clearly display pulmonary infection lesions, improve the clinical diagnosis of severe pneumonia complicated with pulmonary infection, and help to screen early pulmonary infection and carry out symptomatic treatment. However, this study has not solved the unideal segmentation effect of the EC-U-net model on microscopic structures such as tiny pulmonary vessels, and imaging analysis of pulmonary infections caused by different pathogens is lacking. In future studies, we will include more case data of patients with severe pneumonia complicated with pulmonary infection, and conduct more image segmentation experiments with the proposed algorithm to verify the reliability of deep learning technology. In conclusion, the results provide data support for the clinical diagnosis and treatment of severe pneumonia complicated with pulmonary infection.

## Data availability statement

The original contributions presented in this study are included in this article/supplementary material, further inquiries can be directed to the corresponding author.

## Ethics statement

The studies involving human participants were reviewed and approved by the China Academy of Chinese Medical Sciences. The patients/participants provided their written informed consent to participate in this study.

## Author contributions

MM: writing-original draft, conceptualization, and formal analysis. NL: software and validation. WQ: methodology, writing-review, and editing. All authors contributed to the article and approved the submitted version.
